# A computational model of striatal neural microcircuit: how dopamine release becomes important to the striatal functions

**DOI:** 10.1186/1471-2202-14-S1-P353

**Published:** 2013-07-08

**Authors:** Berat Denizdurduran, Neslihan Serap Sengor

**Affiliations:** 1 Electronics and Communications Engineering Department, Istanbul Technical University, Istanbul, 34469, Turkey

## 

The striatum is the main input structure of the basal ganglia circuits and the functions of this subcortical region are modified with dopaminergic innervation released from Substantia Nigra pars Compacta [1]. We have focused on the formation of this subcortical region and its relation to the dopamine neurotransmitter since striatum has a key role in the basal ganglia functions, such as action selection and reward-based learning. The aim of this study is to find a plausible way to explain the effect of dopamine release on the functions and formations of striatal microcircuits. This work also focuses on the pathological synchrony within D2 MSN network which causes the repetitive projections of the Globus Pallidus External and Subthalamic Nucleus network [2]. This pathological synchrony would be related to parkinsonian behavior as this network is primarily responsible for motor behaviors [3]. It is now claimed that structure of the striatum is modified with long term depression (LTD) and long term potentiation (LTP) and dopamine regulates this structure [4]. So, besides examining D2 network, we also have focused on D1 network to be able to explain how increased level of dopamine release causes the new formation within this region. In this work, we are following the Fast-Slow dynamical system approach to be able to model the Medium Spiny neurons. The striatal network comprises of 400 D1 MSNs, 400 D2 MSNs modeled as a conductance-based compartmental neuron model with 20 Fast-spiking inhibitory (FSIN) Adaptive and Exponential neuron models (%2) which matches the experimental observations [2].

**Figure 1:**
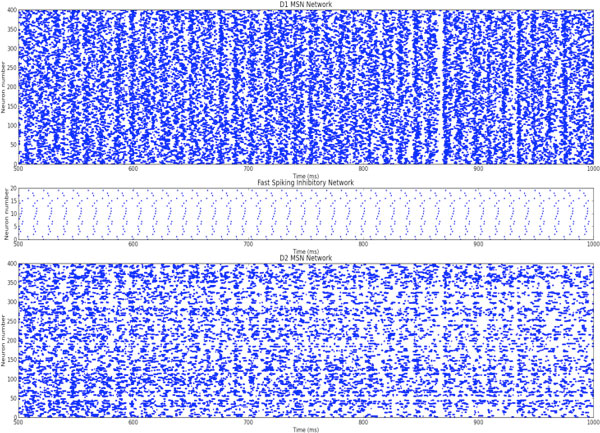
**Raster plot of striatal microcircuit which comprises 400 D1 MSN (top), 20 FSIN (middle) and 400 D2 MSN (bottom)**.

Here, we show that dopamine has the ability to modify both D1 and D2 MSNs as it can be followed in Figure 1. With a biased dopamine release, both network reach stabilize firing regime while increased dopamine causes an overreacted firing pattern in D1 network, it suppresses D2 network firing rate, see Figure 1. In the proposed network FSINs have all-to-all connections to the D1 and D2 MSNs to understand how these feedforward inhibition controls the pathological neuronal oscillations. We have examined the contribution of different dopamine levels to control pathological neuronal oscillations and explained whether Poisson distributed spike trains correlate with dopamine level, significantly.
